# Giant cell reparative granuloma of the calcaneus in a 32-year-old man without recurrence at 7-year follow-up: a case report

**DOI:** 10.3389/fsurg.2026.1859570

**Published:** 2026-08-05

**Authors:** Xiaowei Wang, Diao Yu, Jingjing Liu, Zhengchang Yang, Qiaojun Chen, Li Bian, Juan Chen, Chengyuan Hu

**Affiliations:** 1 Department of Spinal Surgery, Southern Central Hospital of Yunnan Province, Honghe, Yunan, China; 2 Department of Clinical Laboratory, Zhejiang Provincial People’s Hospital Bijie Hospital, Bijie, Guizhou, China; 3 Department of Spinal Surgery, The First Affiliated Hospital of Dali University, Dali, Yunnan, China; 4 Department of Clinical Psychology, Southern Central Hospital of Yunnan Province, Honghe, Yunan, China; 5 Department of Orthopedics, Wusheng Hospital of Traditional Chinese Medicine in Sichuan Province, Guangan, Sichuan, China

**Keywords:** bone grafting, calcaneus, case report, curettage, giant cell reparative granuloma, lytic lesion

## Abstract

**Background:**

Giant cell reparative granuloma (GCRG) is a rare benign lesion, with calcaneal involvement being particularly uncommon. The differential diagnosis of osteolytic lesions in the foot remains challenging due to radiographic overlap with other entities.

**Case report:**

A 32-year-old man presented with a five-year history of recurrent left heel pain that acutely worsened over ten days. Radiography revealed an expansile, well-defined lytic lesion with a sclerotic margin in the posterior calcaneus. The patient underwent curettage and bone grafting. Histopathology demonstrated a proliferative stroma of spindle-shaped fibroblasts and myofibroblasts, accompanied by numerous osteoclast-like giant cells, foamy histiocytes, and focal hemosiderin deposition. Immunohistochemistry was positive for CD68 and EMA, negative for P63 and SMA, and showed a low Ki-67 proliferation index. At 7-year follow-up, the patient remained asymptomatic with no evidence of recurrence.

**Conclusions:**

GCRG of the calcaneus should be considered in the differential diagnosis of osteolytic foot lesions in young adults. Definitive diagnosis relies on histopathological and immunohistochemical examination. Curettage combined with bone grafting is an effective treatment that achieves long-term recurrence-free survival.

## 1 Introduction

The differential diagnosis of an osteolytic lesion in the foot poses a significant clinical challenge because of the radiographic overlap among entities with vastly different prognoses and treatment strategies. These lesions encompass a broad spectrum of conditions, ranging from neoplastic and hematologic disorders to infections and reactive processes. Among them, giant cell reparative granuloma (GCRG) is a rare, benign, non-neoplastic lesion most frequently documented in the jawbones (1). Its occurrence in the extremities is infrequent, and calcaneal involvement is particularly uncommon (2, 3). This case report describes a rare case of GCRG affecting the calcaneus, highlighting the paramount role of histopathology in establishing a definitive diagnosis.

## 2 Case report

**Figure 1:**
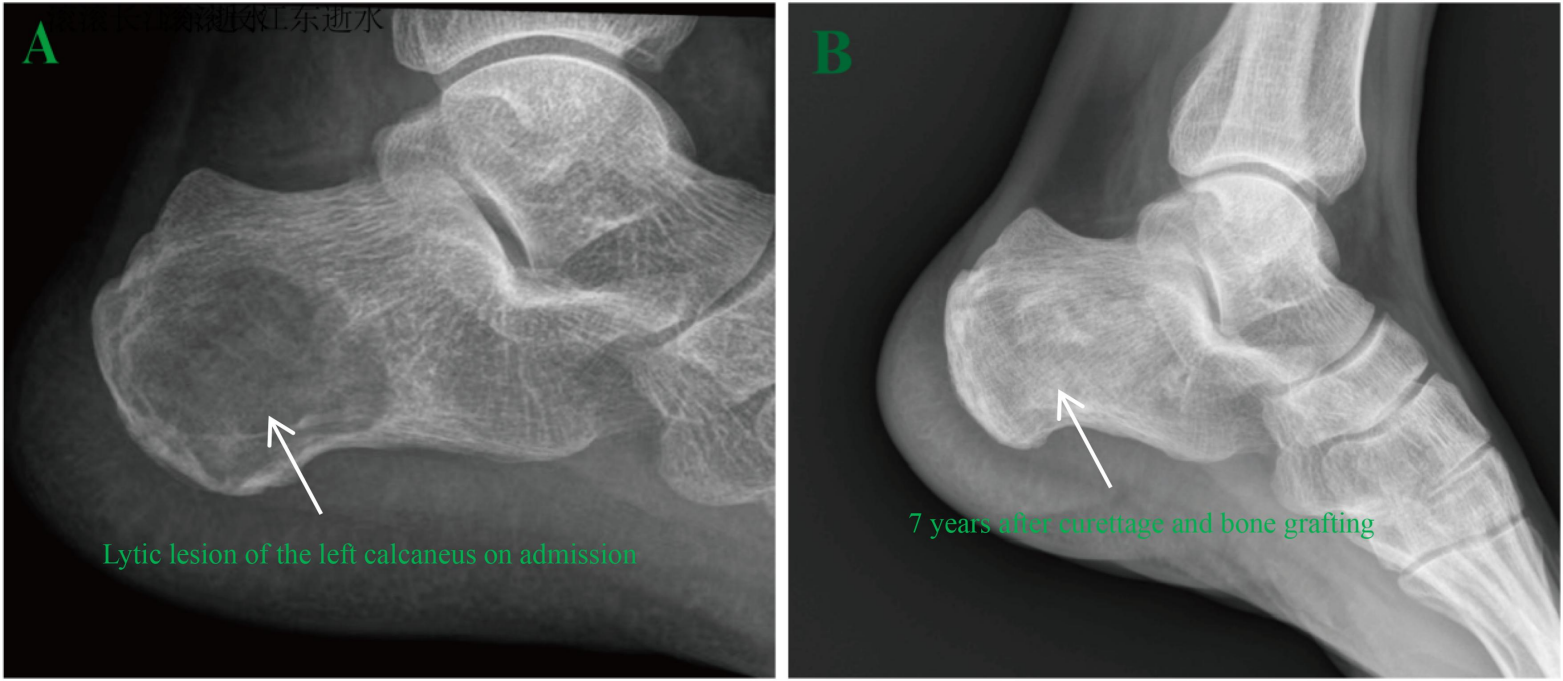
**(A)** A cystic lucency is noted within the posterior aspect of the left calcaneus, surrounded by a sclerotic border on x-ray. The adjacent soft tissue planes are preserved. **(B)** The bone cortex remains continuous, and the postoperative appearance after curettage and bone grafting shows no evidence of recurrence on x-ray at the 7-year follow-up. (Source: Original images obtained from the Department of Radiology).

A 32-year-old man presented with a five-year history of recurrent pain in his left heel. The pain acutely intensified over ten days, resulting in a noticeable limp. He denied any history of significant trauma or systemic illnesses. Physical examination revealed mild swelling and focal tenderness of the left heel. Routine laboratory investigations, including serum levels of calcium, inorganic phosphorus, alkaline phosphatase, and parathyroid hormone, were within normal limits. x-ray showed an expansile, well-defined lytic lesion surrounded by a sclerotic margin in the posterior aspect of the left calcaneus, with unremarkable adjacent soft tissues (Figure 1A). Magnetic resonance imaging (MRI) was unavailable as the patient declined. Preoperative differential diagnoses included simple bone cyst, aneurysmal bone cyst (ABC), and giant cell tumor (GCT). However, the absence of fluid-fluid levels on MRI and the non-epiphyseal location argued against ABC and GCT, prompting surgical intervention for definitive diagnosis.

**Figure 2:**
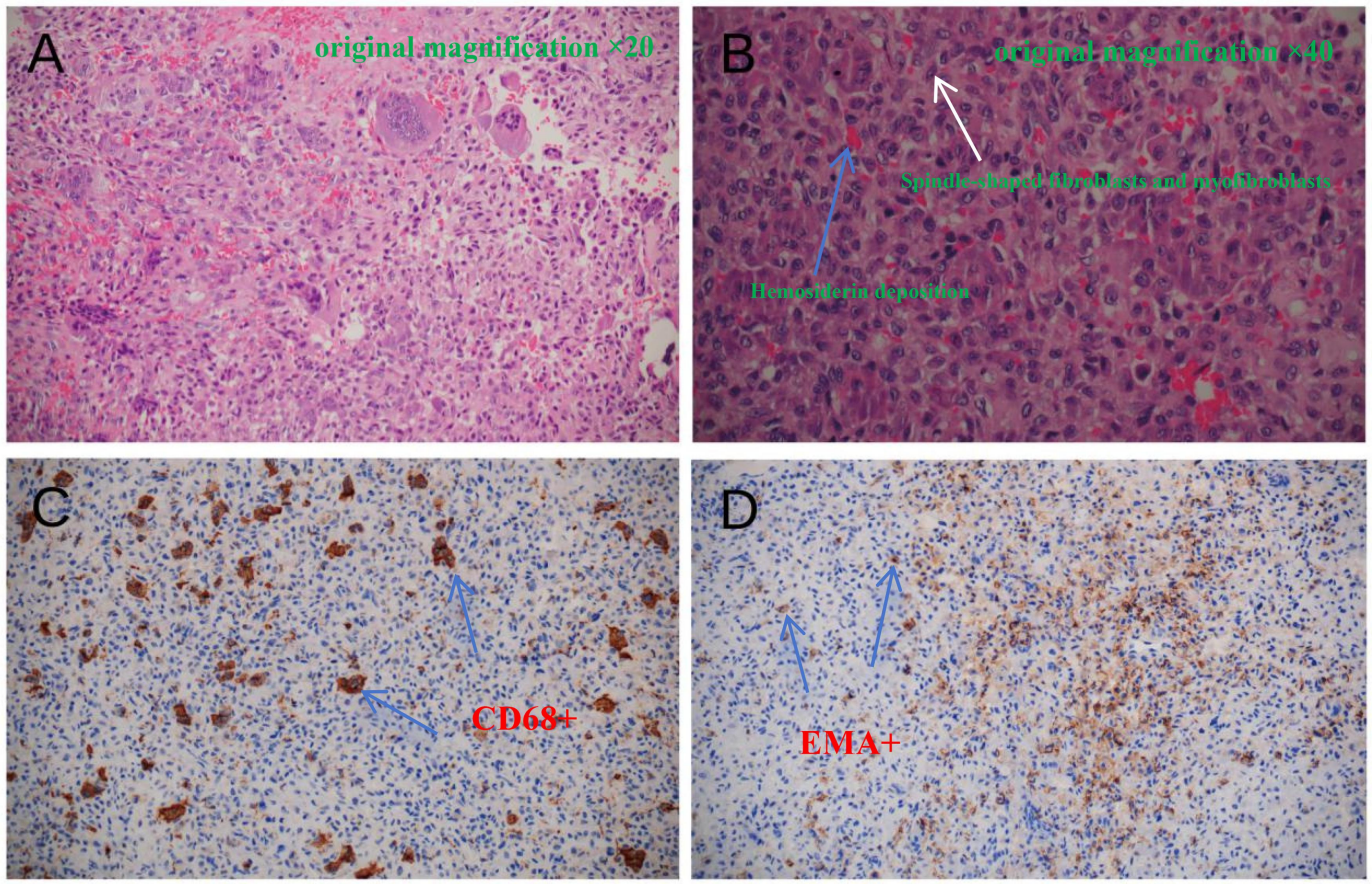
**(A,B)** Spindle-shaped fibroblasts and myofibroblasts, within which numerous osteoclast-like giant cells and foamy histiocytes were scattered. Hemosiderin deposition was also evident within the stroma. [**(A)** Hematoxylin-eosin, original magnification ×20; **(B)** Hematoxylin-eosin, original magnification ×40]. **(C,D)** Immunohistochemical staining was positive for CD68 and EMA, and was negative for P63 and SMA (E: CD68+. F: EMA+). (Source: Original photomicrographs from the Department of Pathology).

The lesion was treated by curettage followed by bone grafting, using a composite of autologous bone harvested from the left iliac crest and allogeneic bone graft material. Intraoperative findings included an intact cortex, a sclerotic medullary cavity wall, and granulomatous-like tissue lining the inner wall. Histopathological examination revealed a proliferative stroma of spindle-shaped fibroblasts and myofibroblasts, with numerous scattered osteoclast-like giant cells and foamy histiocytes. Focal hemosiderin deposition was also observed within the stroma (Figures 2A,B). Immunohistochemical staining was positive for CD68 and EMA, showed a low proliferative index with Ki67, and was negative for P63 and SMA (Figures 2C,D). Although EMA positivity is not typical for giant cell reparative granuloma (GCRG), the overall histomorphological and immunohistochemical features were consistent with this diagnosis after multidisciplinary correlation.

Thus, the patient was ultimately diagnosed with GCRG. During the 7-year follow-up, annual clinical assessments and x-ray examinations were performed. The patient remained asymptomatic with complete resolution of left heel pain and no recurrence at any visit. The final x-ray at 7 years showed continuity of the bone cortex and no evidence of lesion recurrence (Figure 1B).

## 3 Discussion

In the present case, the lesion occurred in the rare location of the calcaneus, and no recurrence was observed after curettage and bone grafting during long-term follow-up.

Although our case had a favorable long-term outcome without recurrence after surgical treatment, the preoperative radiographic finding of an osteolytic lesion in the calcaneus posed a significant diagnostic challenge due to significant overlap in the clinical and histopathological features of these disorders, including giant cell tumor of bone (GCT), aneurysmal bone cyst (ABC), and brown tumor of hyperparathyroidism (4). These entities may all present as expansile, well-circumscribed lytic lesions on imaging, overlapping considerably with the appearance of GCRG, as summarized in Table 1. Therefore, a definitive diagnosis requires comprehensive assessment of clinical presentation, imaging, pathology, anatomic site, patient age, and lesion count.

**Table 1:** Differential diagnosis of lytic lesions of bone.

Terminology	Age (years)	Typical location	Imaging features (CT/MRI)	Histopathology	Laboratory findings	Prognosis
GCRG	20–30	Jawbones (most common); short tubular bones of hands/feet	CT: Expansile, lytic, well-circumscribed lesion with sclerotic margin; cortex thinned but intact.	Patternless fibrous stroma with spindle cells; giant cells clustered around foci of hemorrhage and hemosiderin; no cytologic atypia	Normal serum calcium, phosphorus, ALP, PTH	Benign, non-neoplastic, locally aggressive; recurrence rate 10%–75%
			MRI: Low signal on T1 and T2 weighted images			
GCT	30–40	Epiphyses of long bones (distal femur, proximal tibia)	CT: Purely lytic, eccentric, “soap-bubble” appearance; extends to subchondral bone.	Uniformly distributed giant cells among ovoid to spindle mononuclear stromal cells; nuclei of stromal cells resemble those of giant cells	Normal (unless pathological fracture)	Locally aggressive, benign but can metastasize (rarely to lungs); high recurrence rate after simple curettage
			MRI: Low to intermediate signal on T1WI; intermediate to high signal on T2WI			
ABC	10–20	Metaphysis of long bones or posterior elements of spine	CT: Multiloculated, expansile “blow-out” lytic lesion.	Blood-filled cystic spaces separated by fibrous septa; septa contain fibroblasts, osteoid, and scattered giant cells; no endothelial lining	Normal	Benign, locally destructive; can be primary or secondary; recurrence possible
			MRI: Fluid-fluid levels (characteristic)			
Brown Tumor	30–50	Bones of extremities and large bones (mandible, ribs, sternum)	CT: Lytic, expansile, often multiple lesions; may show generalized osteopenia.	Reactive fibrous tissue with histiocytes, osteoclasts, and osteoclastic giant cells; osteoblastic rimming; histologically indistinguishable from GCRG	Elevated serum calcium, ALP, and PTH; low or normal serum phosphorus	Benign, reactive lesion; resolves with treatment of underlying hyperparathyroidism
			MRI: Iso- or hypointense on T1WI; hyper- or hypointense on T2WI			

Source: Compiled by the authors based on data from published literature [references (2, 4, 5, 7–9)].

Once diagnosed, the GCRG lesion should be completely removed whenever possible. While GCRG is a benign non-neoplastic lesion, its postoperative recurrence rate cannot be overlooked. Ari I et al. reported the recurrence rates were 21.4% in peripheral giant cell granuloma and 33.3% in central giant cell granuloma (5). Similarly, Somit Mittal et al. found that recurrence rate was still observed in 25% to 60% of cases after 6 months of follow-up, despite attempts at lesion eradication (6). However, a recent study by Gao et al. revealed four cases of GCRG with a follow-up of 2 to 3 years, all of which remained recurrence-free (7), a finding consistent with our study, in which no recurrence was observed over a 7-year follow-up period. It is noteworthy that the high recurrence rates were reported in earlier studies, whereas more recent studies have shown lower rates. This discrepancy may be attributable to relatively delayed diagnosis and less advanced surgical techniques in the past, leading to incomplete removal and subsequent recurrence. Moreover, Aliu F et al. found that conservative treatments, including intralesional corticosteroids, calcitonin, interferon-alpha, and denosumab, have been suggested to potentially yield lower recurrence rates than surgical excision and curettage (8). Thus, aggressive and thorough curettage combined with bone grafting represents a safe and effective treatment option for GCRG, achieving long-term recurrence-free survival.

Immunohistochemically, EMA is not expected to be expressed in GCRG; however, in the present case, focal positivity for EMA was observed. Importantly, the overall histomorphological features, including a patternless fibrous stroma, clustered giant cells around areas of hemorrhage, and hemosiderin deposition, were entirely consistent with GCRG. Furthermore, the patient had no recurrence during the 7-year follow-up period, and negative staining for P63 and SMA helped exclude the possibility of GCT.

Several limitations should be acknowledged. First, this is a single case report, which limits generalizability, and late recurrence beyond this period cannot be entirely excluded despite the 7-year follow-up. Second, we did not perform molecular studies (e.g., USP6 rearrangement testing), which could have helped distinguish GCRG from primary ABC, as USP6 rearrangements are characteristic of ABC but absent in GCRG (9). Third, a preoperative needle biopsy was not performed to characterize the lesion before surgical curettage. While the imaging features and clinical course were strongly suggestive of a benign process, we recognize that histopathological confirmation prior to definitive surgery would have been preferable, especially given the unusual location. In future similar cases, we would consider performing a preoperative closed needle biopsy to guide surgical planning.

## 4 Conclusion

GCRG of the calcaneus is an extremely rare entity that should be considered in the differential diagnosis of osteolytic lesions of the foot in young adults. Given the significant radiographic overlap with other giant cell-rich lesions such as GCT and ABC, a definitive diagnosis relies on histopathological and immunohistochemical examination. Surgical curettage combined with bone grafting remains an effective treatment option to achieve long-term recurrence-free survival.

## Data Availability

The raw data supporting the conclusions of this article will be made available by the authors, without undue reservation.
